# Infant and young child feeding practices and child linear growth in Nepal: Regression–decomposition analysis of national survey data, 1996–2016

**DOI:** 10.1111/mcn.12911

**Published:** 2020-01-10

**Authors:** Giles Hanley‐Cook, Alemayehu Argaw, Pradiumna Dahal, Stanley Chitekwe, Patrick Kolsteren

**Affiliations:** ^1^ Department of Food Technology, Safety and Health, Faculty of Bioscience Engineering Ghent University Ghent Belgium; ^2^ Department of Population and Family Health, Institute of Health Jimma University Jimma Ethiopia; ^3^ Nutrition Section, United Nations Children's Fund (UNICEF) Kathmandu Nepal

**Keywords:** breastfeeding, complementary feeding, infant and young child feeding, Nepal, statistical decompositions, stunting, undernutrition

## Abstract

Suboptimal infant and young child feeding (IYCF) practices have profound implications on child survival, health, growth, and development. First, our study analysed trends in 18 IYCF indicators and height‐for‐age z‐score (HAZ) and stunting prevalence across Nepal's Family Health Survey 1996 and four rounds of Nepal Demographic and Health Surveys from 2001–2016. Second, we constructed multivariable regression models and decomposed the contribution of optimal IYCF practices on HAZ and stunting prevalence over the 1996–2016 period. Our findings indicate that most age‐appropriate IYCF practices and child linear growth outcomes improved over the past two decades. At present, according to the World Health Organization's tool for national assessment of IYCF practices, duration of breastfeeding is rated *very good*, early initiation of breastfeeding and exclusive breastfeeding (EBF) are rated *good*, whereas minimal bottle‐feeding and introduction of solid, semi‐solid or soft foods are rated *fair*. Our study also reports that a paucity of age‐appropriate IYCF practices—in particular complementary feeding—are significantly associated with increased HAZ and decreased probability of stunting (*p* < .05). Moreover, age‐appropriate IYCF practices—in isolation—made modest statistical contributions to the rapid and sustained reduction in age‐specific child linear growth faltering from 1996–2016. Nevertheless, our findings indicate that comprehensive multisectoral nutrition strategies—integrating and advocating optimal IYCF—are critical to further accelerate the progress against child linear growth faltering. Furthermore, specific focus is needed to improve IYCF practices that have shown no significant development over the past two decades in Nepal: EBF, minimum acceptable diet, and minimal bottle‐feeding.

**Key Messages**

- Nepal made substantial progress on infant and young child feeding (IYCF) practices and linear growth outcomes from 1996–2016.
- A paucity of IYCF indicators—in particular complementary feeding practices—are significantly associated with height‐for‐age z‐score and stunting prevalence.
- Age‐appropriate IYCF practices—in isolation—made modest statistical contributions to the rapid and sustained reduction in age‐specific child linear growth faltering over the past two decades.
- Nevertheless, the integration and monitoring of IYCF policies, programmes, health systems, workplaces, and communities remains critical to further accelerate the progress against chronic child undernutrition in Nepal.

## 1 INTRODUCTION

Child malnutrition is a major public health concern worldwide. At present, an estimated 149 million children under five are stunted, whereas the lives of over 49 million children continue to be threatened by wasting (UNICEF, WHO [World Health Organization], & World Bank Group, 2019). These malnourished children are at a higher risk of mortality and poor health, growth, and development (Black et al., 2008). The causes of child linear growth faltering are complex, multidimensional, and interrelated. UNICEF's (1991) framework, updated by Black et al. (2013), propagates that nutrition outcomes are the end result of a causal chain of determinants. Among the most proximal and immediate determinants are suboptimal infant and young child feeding (IYCF) practices. Optimal IYCF is essential for child nutrition, linear growth, and cognitive development (Victora et al., 2016; WHO, 2008, 2010). An estimated 14% of deaths in children aged 0–23 months might be prevented by scaling up breastfeeding to universal levels, and at least 6% of mortality in children under five might be prevented by adequate complementary feeding (Black et al., 2013; Victora et al., 2016). Furthermore, age‐appropriate IYCF practices are directly linked to the attainment of at least four of the United Nation's Sustainable Development Goals: nutrition, health, poverty reduction, and inequity reduction (Rollins et al., 2016).

In South Asia, at least one in three children under five is stunted (34.4%), and it is the only region with a very high wasting prevalence (15.2%; UNICEF et al., 2019). Over the past two decades, South Asian nations—in particular Nepal—have achieved unprecedented progress on reducing chronic child undernutrition (Cunningham, Headey, Singh, Karmacharya, & Rana, 2017; Headey, Hoddinott, & Park, 2017). However, recent research has reported that maternal and caregiver IYCF knowledge is suboptimal (Cunningham, Headey, et al., 2017; Senarath et al., 2012), and most breastfeeding (Benedict, Craig, Torlesse, & Stoltzfus, 2018) and complementary feeding practices (Na et al., 2018) have improved only marginally in recent years.

In this study, we address the research gap with regard to long‐term trends in age‐appropriate IYCF practices and their contribution to the rapid and sustained progress against child linear growth faltering in Nepal. We aim to answer three interrelated questions: (a) What are the long‐term trends in 18 IYCF indicators, height‐for‐age z‐score (HAZ) and stunting prevalence in Nepal? (b) which IYCF indicators are associated with age‐specific HAZ and stunting? and (c) which IYCF practices potentially account for variations in child linear growth faltering observed from 1996–2016? We also briefly discuss the policy and programme implications of our findings to inform actions to protect, promote, and support IYCF practices in Nepal.

## 2 METHODS

### 2.1 Data sources

The estimates reported in this research paper were constructed using individual child‐level data from the following cross‐sectional surveys: Nepal's Family Health Survey (NFHS) 1996 and four rounds of Nepal Demographic and Health Survey (NDHS) 2001, 2006, 2011, and 2016. These data are well suited to our research, as the five surveys are high quality, nationally representative, standardised across rounds and cover a broad—albeit non‐exhaustive—range of hypothesised nutrition‐sensitive and nutrition‐specific determinants of child linear growth and anthropometric measurements.

### 2.2 Outcomes

HAZ and stunting (HAZ < −2 SD) were measured against the median of the WHO (2006) Child Growth Standard. Linear growth is generally regarded as the single most relevant indicator of overall child nutrition status, and the reduction of stunting is the standard metric of long‐term nutritional, educational, and economic progress (Dewey & Begum, 2011; Hoddinott et al., 2013).

### 2.3 Independent variables

Table 1 presents 18 IYCF indicators constructed according to WHO (2008, 2010) and UNICEF (2016) recommendations. There are eight core IYCF indicators: early initiation of breastfeeding within 1 hr of birth (EIBF); exclusive breastfeeding under 6 months (EBF); continued breastfeeding at 1 year; introduction of solid, semi‐solid or soft foods at 6–8 months (INTRO); and minimum dietary diversity (MDD); minimum meal frequency; minimum acceptable diet (MAD); and consumption of iron‐rich or iron‐fortified foods (IRON) in children 6–23 months. In addition, there are seven optional indicators: children ever breastfed; continued breastfeeding at 2 years; age‐appropriate breastfeeding; predominant breastfeeding under 6 months; median duration of breastfeeding; bottle‐feeding; and milk feeding frequency for non‐breastfed children. Our analysis also examined three additional indicators: avoidance of prelacteal feeding in the first 3 days following delivery, optimal early breastfeeding, and consumption of animal‐source foods (ASF).

**Table 1:** Indicators for infant and young child feeding practices

Indicators	Description
Core indicators	
1. Early initiation of breastfeeding	Proportion of children born in the last 24 months who were put to the breast within 1 hr of birth
2. Exclusive breastfeeding under 6 months	Proportion of infants 0–5 months of age who are fed exclusively with breast milk
3. Continued breastfeeding at 1 year	Proportion of children 12–15 months of age who are fed breast milk
4. Introduction of solid, semi‐solid, or soft foods	Proportion of infants 6–8 months of age who receive solid, semi‐solid, or soft foods
5. Minimum dietary diversity	Proportion of children 6–23 months of age who receive foods from four or more out of seven food groups
6. Minimum meal frequency	Proportion of breastfed and non‐breastfed children 6–23 months of age who receive solid, semi‐solid, or soft foods (including milk feeds for non‐breastfed children) the minimum number of times or more
7. Minimum acceptable diet	Proportion of children 6–23 months of age who receive a minimum acceptable diet (apart from breast milk)
8. Consumption of iron‐rich or iron‐fortified foods	Proportion of children 6–23 months of age who receive an iron‐rich or iron‐fortified food that is specially designed for infants and young children, or that is fortified in the home
Optional indicators	
9. Children ever breastfed	Proportion of children born in the last 23 months who were ever breastfed
10. Continued breastfeeding at 2 years	Proportion of children 20–23 months of age who are fed breast milk
11. Age‐appropriate breastfeeding	Proportion of children 0–23 months of age who are appropriately breastfed
12. Predominant breastfeeding under 6 months	Proportion of infants 0–5 months of age who are predominantly breastfed
13. Duration of breastfeeding	Median duration of breastfeeding among children 0–35 months of age
14. Bottle‐feeding	Proportion of children 0–23 months of age who are fed with a bottle
15. Milk feeding frequency for non‐breastfed children	Proportion of non‐breastfed children 6–23 months of age who receive at least two milk feedings (infant formula, cow's milk or other animal milk)
Additional indicators	
16. No prelacteal feeding	Proportion of children born in the last 24 months who did not receive anything other than breastmilk in the first 3 days
17. Optimal early breastfeeding	Proportion of children born in the last 24 months were put to the breast within 1 hr of birth and did not receive anything other than breastmilk in the first 3 days
18. Consumption of animal‐source foods	Proportion of children 6–23 months of age who receive one or more animal‐source food type: (a) dairy; (b) egg; (3) flesh foods

*Note*. Indicators 2–8, 10–12, and 14–15 are based on a 24‐hr recall period. Indicators 1, 2, 7, and 8 are considered top priorities for reporting among the core indicators. Indicator 2 can be disaggregated for ages 0–1, 2–3, 4–5, and 0–3 months. The seven food groups mentioned under indicator 5 are grains, roots, and tubers; legumes and nuts; dairy products (milk, yogurt, cheese); flesh foods (meat, fish, poultry, and liver/organ meats); eggs; vitamin A‐rich fruits and vegetables; other fruits and vegetables. Minimum number of times mentioned under indicator 6 is defined as: two times for breastfed infants 6–8 months; three times for breastfed children 9–23 months; four times for non‐breastfed children 6–23 months. Indicator 7 is the sum of two fractions: (1) the proportion of breastfed children 6–23 months of age who had at least the minimum dietary diversity and the minimum meal frequency during the previous day; plus (2) the proportion of non‐breastfed children 6–23 months of age who received at least two milk feedings and had at least the minimum dietary diversity and the minimum meal frequency during the previous day. Indicator 11 is the sum of exclusive breastfeeding under 6 months plus the proportion of children 6–23 months of age who received breast milk as well as solid, semi‐solid, or soft foods during the previous day.

Furthermore, covariates from child‐, parental‐, and household‐level were selected based on the Black et al. (2013) framework and a review of previous regression–decomposition analyses of chronic child undernutrition (Cunningham, Headey, et al., 2017; Headey et al., 2017; Headey & Hoddinott, 2015; Headey, Hoddinott, Ali, Tesfaye, & Dereje, 2015; Headey, Hoddinott, & Park, 2016; Menon, Headey, Avula, & Nguyen, 2018). These time‐variant independent variables included a number of hypothesised nutrition‐sensitive and nutrition‐specific determinants of child linear growth faltering, namely, household asset index (Filmer & Pritchett, 2001), maternal education, paternal education, prenatal doctor visits, 4^+^ antenatal care visits, iron during pregnancy, childbirth in a medical facility, maternal body mass index and height, child vaccination, birth order, birth interval, improved household sanitation, and drinking water source (Table S1). Our multivariable regression models also included time‐invariant control variables, namely, dummy variables for child age (months), child sex, region, agroecological zone, religion, ethnicity, maternal age (5‐year intervals), and survey rounds.

### 2.4 Statistical analysis

Data management and statistical analysis were conducted in Stata Version 15.1 (StataCorp, 2017). First, we examined long‐term trends in age‐appropriate IYCF practices, HAZ, and stunting prevalence among various age groups of children—between 0 to 35 months—across five national surveys in Nepal. Summary statistics include population‐adjusted survey weights to account for the multistage cluster sampling design of NFHS 1996 and NDHS 2001–2016. Our analysis excluded observations with HAZ beyond the range of ±6 SD from the median.

In contrast to previous studies, our research applied a quantitative and dynamic regression–decomposition approach, which included IYCF indicators as determinants of child linear growth faltering. Our statistical decompositions comprised two distinct steps: (a) use multivariable regression models—ordinary least squares (OLS) for HAZ and linear probability model for stunting—to assess age‐specific associations (*β*‐coefficients) between age‐appropriate IYCF practices and linear growth outcomes among Nepalese children and (b) decompose the potential contribution of age‐appropriate IYCF practices on age‐specific progress against chronic child undernutrition over the 1996–2016 period. Independent variables with variance inflation factors ≥ 4 were omitted from our regression models to avoid potential collinearity. *p* values < .05 were considered statistically significant for associations between IYCF indicators and child linear growth outcomes.

Our multivariable regression models are represented in Equation (1) below, assessing the associations between linear growth outcomes (*N*) for a child *i* at time *t* and vectors of IYCF indicators (*X*), vectors of time‐variant nutrition‐sensitive and nutrition‐specific determinants (*μ*_*v*_), vectors of mainly time‐invariant control variables (*μ*_*i*_), trend effects represented by a vector of year dummy variables (*T*), and a standard error term (*ε*_*i,t*_).

(1) $$N_{i,t}={\mathrm{\beta X}}_{i,k}+\mu_{v}+\mu_{i}+T+\varepsilon_{i,t}$$

In a simple decomposition at means described in Equation (2), in which *β*‐coefficients are assumed to be stable over time, the estimated contribution of an IYCF indicator (*X*) on a linear growth outcome (*N*) is the product of its *β*‐coefficient and the change in its mean over time. Hence, an IYCF indicator will make a substantial contribution if its *β*‐coefficient is large and if its mean score changes considerably over time. For our analysis, we selected the earliest (NFHS 1996) round (*t* = 1) and the most recent (NDHS 2016) round (*t = k*) available.

(2) $$\Delta {\bar{N}}_{i,t}=\beta \left({\bar{X}}_{t=k}-{\bar{X}}_{t=1}\right),$$

To assess the assumption of stable IYCF *β‐*coefficients, we conducted an Oaxaca–Blinder decomposition testing for systematic differences in *β‐*coefficient between the NFHS 1996 and NDHS 2016 rounds (Jann, 2008). Furthermore, we also estimated models that excluded potentially endogenous determinants of optimal IYCF, including wealth, parental education, and healthcare‐related service variables. Lastly—to determine potential attenuation of *β*‐coefficients by younger children—we tested the associations between age‐appropriate complementary feeding practices and linear growth outcomes for children aged 18–23 months (Alderman & Headey, 2018).

Our research paper did not aim to assess the multifactorial determinants of optimal IYCF and child linear growth in Nepal (Benedict et al., 2018; Headey et al., 2017; Na et al., 2018), nor emulate the detailed definitions, implications, and rationales behind the WHO and UNICEF IYCF indicators (Jones et al., 2014; UNICEF, 2016; WHO, 2008, 2010).

## 3 RESULTS

### 3.1 Trends in age‐appropriate IYCF practices

Table 2 presents the trends in 18 age‐specific IYCF indicators between the NFHS 1996 and NDHS 2016 rounds. Our findings indicate a 6.9% reduction in EBF prevalence. Nevertheless, the *good* WHO tool rating—for the national assessment of IYCF practices (WHO, 2003)—remained unchanged over the past two decades (50–89%), and Nepal remains above the World Health Assembly 2025 EBF target of 50%. In parallel, INTRO improved by 22.8 percentage points (p.p.) from 1996–2016. This substantial increase translated into a change in the WHO tool rating for INTRO from *poor* (0–59%) to *fair* (60–79%). Furthermore, EIBF increment of 38 p.p.—from a low initial base of 18.1% in 1996—resulted in a transition from a *poor* (0–29%) to a *good* (50–89%) rating according to the WHO tool. MDD increased by 14.0 p.p. from 30.5% in 2006 and IRON increased by 18.1 p.p. over the last decade, from only 20.8% in 2006. In contrast, an undesirable 11.3 p.p. increment in bottle‐feeding was reflected in a diminished WHO tool rating from *good* (3–4%) to *fair* (5–29%). The population‐level median duration of breastfeeding increased by 4 months over the 1996–2016 period to 35 months in 2016. The high initial base in 1996 and gradual increment is reflected in the persistent *very good* (≥23–24 months) WHO tool rating. The avoidance of prelacteal feeding increased by 11.8 p.p. from 59.5% in 2001. Nevertheless, one in four children (28.7%) are still provided with prelacteal feeds, which presents a significant challenge to achieve the WHO 90% EBF recommendation. Achieving both EIBF and avoidance of prelacteal feeding—coined as optimal early breastfeeding—improved by 65.7%, from a low initial base of 27.1% in 2001.

**Table 2:** Trends in infant and young child feeding practices, 1996–2016

Infant and Young Child Feeding (IYCF) indicators	1996	2001	2006	2011	2016	Change	Percent change (%)	N
Core indicators								
1. Early initiation of breastfeeding (%)	18.1	30.8	35.9	45.7	55.7	37.6	207.7	11,350
2. Exclusive breastfeeding under 6 months (%)	71	—	50.4	67.1	66.1	−4.9	−6.9	2,219
3. Continued breastfeeding at 1 year (%)	90.5	98.5	92.3	98.4	86.9	−3.6	−4	2,014
4. Introduction of solid, semi‐solid, or soft foods (%)	54.3	—	64	61.3	77.1	22.8	42	1,158
5. Minimum dietary diversity (%)	—	—	30.5	27.3	44.5	14	45.9	4,506
6. Minimum meal frequency (%)	65.8	67.6	82.5	77.8	70.2	4.4	6.9	7,956
7. Minimum acceptable diet (%)	—	—	27.6	22.7	33.7	6.1	22.1	4,453
8. Consumption of iron‐rich or iron‐fortified foods (%)	—	—	20.8	25.4	38.9	18.1	87	4,491
Optional indicators								
9. Children ever breastfed (%)	93	97.8	93.6	95.6	95.3	2.3	2.5	11,645
10. Continued breastfeeding at 2 years (%)	84.8	90.3	83.9	96.9	88.7	3.9	4.6	1,916
11. Age‐appropriate breastfeeding (%)	75.7	—	75.4	77.6	82.4	6.7	8.9	9,078
12. Predominant breastfeeding under 6 months (%)	82.4	—	84.7	87.1	86.6	4.2	5.1	2,219
13. Median duration of breastfeeding (months)	31	34	34	34	35	4	11.4	16,509
14. Bottle‐feeding (%)	2.8	2.7	3.5	5.7	13.5	10.7	382.1	11,146
15. Milk feeding frequency for non‐breastfed children (%)	—	66.9	—	—	—	—	—	45
Additional indicators								
16. No prelacteal feeding (%)	—	59.5	63.7	75.7	71.3	11.8	19.8	8,285
17. Optimal early breastfeeding (%)	—	27.1	24.4	37.5	44.9	17.8	65.7	8,442
18. Consumption of animal‐source foods (%)	—	—	32	28.9	42	10	31.3	6,510

### 3.2 Trends in child linear growth outcomes

Table 3 reports the long‐term trends HAZ and stunting prevalence for age‐specific samples used to construct the 18 IYCF indicators in Table 2. Our findings indicate rapid and sustained improvements in HAZ and stunting across all age groups from 1996–2016. To illustrate, over the past two decades, children aged 0–5 months attained a 0.81 SD increase in HAZ and 16.5 p.p. reduction in stunting prevalence. In percentage terms, the changes in stunting prevalence were usually larger than the changes in HAZ. Figure S1 presents the distribution of HAZ for children aged 0–23 months in the NFHS 1996 and NDHS 2016 rounds and confirms a larger shift in the lower tail. The prevalence of stunting fell by more than 40% in children 0–23 months between 1996 and 2016 (Table 3).

**Table 3:** HAZ and stunting prevalence by age group, 1996–2016

	HAZ							Stunting (%)						
Samples (months)	0–5	0–23	0–35	6–8	6–23	12–15	20–23	0–5	0–23	0–35	6–8	6–23	12–15	20–23
1996	−1.36	−1.89	−2.18	−1.35	−2.05	−1.95	−2.68	30.2	47.7	56.6	30.9	53.2	50.3	73.5
2001	−1.07	−1.74	−2.01	−1.32	−1.96	−1.93	−2.44	22.2	41.8	50.4	27.9	48.3	47.6	63.2
2006	−0.62	−1.41	−1.71	−1.03	−1.67	−1.63	−2.15	11.6	32.7	41.9	18.2	39.5	36.8	55.1
2011	−0.85	−1.17	−1.47	−0.65	−1.27	−1.11	−1.75	20.0	26.3	35.0	17.6	28.2	24.5	44.8
2016	−0.55	−1.11	−1.34	−0.64	−1.27	−1.34	−1.80	13.7	26.7	32.5	15.6	30.4	30.4	42.4
Change	0.81	0.78	0.84	0.71	0.78	0.61	0.88	−16.5	−21.0	−24.1	15.3	−22.8	−19.9	−31.1
Percent change (%)	59.6	41.3	38.5	52.6	38.0	31.3	32.8	−54.6	−44.0	−42.6	−49.5	−42.9	−39.6	−42.3
*N*	2,139	8,887	13,262	1,154	6,748	1,550	1,467	2,139	8,887	13,262	1,154	6,748	1,550	1,467

*Note*. Stunting (%) refers to HAZ < −2 SD.

Abbreviation: HAZ, height‐for‐age z‐score.

### 3.3 Regression–decomposition

Table 4 reports our age‐specific multivariable regression results for pooled samples of available NFHS and NDHS data rounds. Column 4 reports OLS models of HAZ and column 5 reports linear probability models of stunting against IYCF indicators listed in Table 1, including a range of omitted hypothesised nutrition‐sensitive and nutrition‐specific determinants of child undernutrition and time‐invariant control variables. Our models are used to derive answers to two related, but distinct research questions: (a) How do age‐appropriate IYCF practices associate with HAZ and stunting in Nepalese children, and (b) how much of the changes in age‐specific child linear growth faltering is explained by IYCF practices over the past two decades in Nepal? The estimated *β*‐coefficients are reported in Table 4.

**Table 4:** Infant and young child feeding practices and child linear growth outcomes in pooled regression models

Dependent variable			HAZ (SE)	Stunting (SE)	
Estimator	Survey rounds	Age (months)	OLS	LPM	N
Core indicators					
1. Early initiation of breastfeeding	1996–2016	0–23	0.062* (0.033)	−0.015 (0.012)	7,993
2. Exclusive breastfeeding	1996, 2006–2016	0–5	−0.130 (0.102)	0.040 (0.028)	1,346
3. Continued breastfeeding at 1 year	1996–2016	12–15	0.075 (0.261)	−0.116 (0.079)	1,418
4. Introduction of solid, semi‐solid or soft foods	1996, 2006–2016	6–8	0.334*** (0.109)	−0.041 (0.038)	781
5. Minimum dietary diversity	2006–2016	6–23	0.164*** (0.052)	−0.065*** (0.020)	2,681
6. Minimum meal frequency	1996–2016	6–23	0.095** (0.037)	−0.034** (0.014)	5,963
7. Minimum acceptable diet	2006–2016	6–23	0.148** (0.057)	−0.049** (0.021)	2,661
8. Consumption of iron‐rich or iron fortified foods	2006–2016	6–23	0.102* (0.057)	−0.030 (0.020)	2,661
Optional indicators					
9. Children ever breastfed	1996–2016	0–23	−0.334 (0.364)	0.045 (0.058)	7,993
10. Continued breastfeeding at 2 years	1996–2016	20–23	0.074 (0.125)	0.016 (0.051)	1,328
11. Age‐appropriate breastfeeding	1996, 2006–2016	0–23	0.097* (0.050)	−0.011 (0.016)	5,866
12. Predominant breastfeeding	1996, 2006–2016	0–5	0.238** (0.120)	−0.053 (0.039)	1,346
14. Bottle‐feeding	1996–2016	0–23	−0.125* (0.074)	0.046* (0.024)	7,993
15. Milk feeding frequency for non‐breastfed children	2001	6–23	−0.440 (1.529)	0.365 (0.498)	35
Additional indicators					
16. Avoidance of prelacteal feeding	2001–2016	0–23	0.073* (0.044)	−0.008 (0.016)	5,581
17. Optimal early breastfeeding	2001–2016	0–23	0.063 (0.044)	−0.017 (0.015)	5,581
18. Consumption of animal‐source foods	2006–2016	6–23	0.131** (0.052)	−0.042** (0.019)	2,681

*Note*. Clustered robust standard errors are reported in parentheses. Stunting (%) refers to HAZ < −2 SD. The regressions include a number of time‐variant independent variables, including household asset index, maternal education, paternal education, prenatal doctor visits, four or more antenatal care visits, iron during pregnancy, born in a medical facility, maternal BMI, maternal height, vaccinations status, birth order, birth interval, open defecation, drinking water source, and time‐invariant controls, including regional and agroecological fixed effects for seven groups, an urban dummy, dummy variables for religion and ethnicity, dummy variables for various maternal age groups (in 5‐year intervals), dummy variables of child age (monthly), a child sex dummy and Family Health Survey and Demographic and Health Surveys round dummy variables (Table S1).

Abbreviations: HAZ, height‐for‐age z‐score; SE, standard error; OLS, ordinary least squares; LPM, linear probability model

* Significant at the 10% level.

** Significant at the 5% level.

*** Significant at the 1% level.

Our findings indicate that various IYCF indicators significantly (*p* < .05) predict an improved HAZ, including INTRO (*β* [SE]: 0.33 [0.11]; *p* = .002), MDD (*β* [SE]: 0.16 [0.05]; *p* = .002), minimum meal frequency (*β* [SE]: 0.10 [0.04]; *p* = .010), MAD (*β* [SE]: 0.15 [0.06]; *p* = .010), predominant breastfeeding (*β* [SE]: 0.24 [0.12]; *p* = .049), and consumption of ASF (*β* [SE]: 0.13 [0.05]; *p* = .013). Furthermore, MDD (*β* [SE]: −0.07 [0.02]; *p* = .001), minimum meal frequency (*β* [SE]: −0.03 (0.01); *p* = .013), MAD (*β* [SE]: −0.05 (0.02); *p* = .018), and consumption of ASF (*β* [SE]: −0.04 (0.02); *p* = .030) are also significantly associated with a decreased probability of stunting.

The results above answered the question of how 18 IYCF indicators associate with child linear growth outcomes in Nepal over time (Table 4). Our research now turned to the second question: Which of these IYCF practices appear to have driven the changes in chronic child undernutrition over the 1996–2016 period of rapid and sustained progress? We used the results from Table 2 and Table 4 to investigate to which extent age‐appropriate IYCF practices are potential drivers of change in child linear growth faltering over time. To comprehend how our statistical decompositions in Table 5 are derived, consider the second row of column 4 that reports the predicted change in HAZ, which is the mean change in EIBF (0–23 months) from 1996–2016 in Table 2 (37.6 p.p.) multiplied by the *β*‐coefficient of EIBF on HAZ from our OLS regression in Table 4 (0.062).

**Table 5:** Decompositions of the predicted changes in child growth outcomes

Dependent variable	Survey rounds	Age (months)	HAZ	Stunting (%)
Core indicators				
1. Early initiation of breastfeeding	1996–2016	0–23	0.02	−0.6
2. Exclusive breastfeeding	1996, 2006–2016	0–5	0.01	−0.2
3. Continued breastfeeding at 1 year	1996–2016	12–15	0.00	0.4
4. Introduction of solid, semi‐solid, or soft foods	1996, 2006–2016	6–8	0.08	−0.9
5. Minimum dietary diversity	2006–2016	6–23	0.02	−0.9
6. Minimum meal frequency	1996–2016	6–23	0.00	−0.1
7. Minimum acceptable diet	2006–2016	6–23	0.01	−0.3
8. Consumption of iron‐rich or iron fortified foods	2006–2016	6–23	0.02	−0.5
Additional indicators				
9. Children ever breastfed	1996–2016	0–23	−0.01	0.1
10. Continued breastfeeding at 2 years	1996–2016	20–23	0.00	0.1
11. Age‐appropriate breastfeeding	1996, 2006–2016	0–23	0.01	−0.1
12. Predominant breastfeeding	1996, 2006–2016	0–5	0.01	−0.2
14. Bottle‐feeding	1996–2016	0–23	−0.01	0.5
15. Milk feeding frequency for non‐breastfed children	2001	6–23	−	−
Additional indicators				
16. No prelacteal feeding	2001–2016	0–23	0.01	−0.1
17. Optimal early breastfeeding	2001–2016	0–23	0.01	−0.3
18. Consumption of animal‐source foods	2006–2016	0–23	0.01	−0.4

*Note*. Predicted nutritional change is based on a linear decomposition at means, in which changes in the mean of an age‐specific IYCF indicator (Table 2) is multiplied by the corresponding adjusted *β*‐coefficient (Table 4). Stunting (%) refers to HAZ < −2 SD. Actual changes in child linear growth outcomes are reported in Table 3.

Abbreviation: HAZ, height‐for‐age z‐score.

Our findings in Table 5 indicate that INTRO contributed 0.08 (11.3%) SD to the increase in HAZ (0.71 SD) and −0.9 (5.9%) p.p. to the total reduction in stunting prevalence (−15.3 p.p.) in children aged 6–8 months over the past two decades. MDD and IRON both contributed 0.02 (5.0%) SD to 0.40 SD increment in HAZ in children aged 6−23 months between 2006 and 2016. MDD contributed −0.9 (8.1%) p.p. to the total decrease in stunting prevalence (−8.9 p.p.), whereas IRON contributed −0.5 p.p. (5.6%).

Our multivariable regression models performed less well (larger standard errors) in the NDHS 2016 round when linear growth outcomes were measured for a smaller sample of children aged 0–35 months, that is, every second household compared with the NFHS 1996. We argue that the smaller sample size in the NDHS 2016 round produced this instability, rather than any genuine changes in the *β*‐coefficients of IYCF variables. Nevertheless, Oaxaca–Blinder decomposition did not change the results in Table 5. Our robustness checks excluding household asset index, maternal and paternal education, prenatal doctor visits, and 4^+^ antenatal care visits from the regression models, did not significantly change the IYCF *β*‐coefficients. Our multivariable regressions—of complementary feeding practices—in children aged 18–23 months reported a higher, but nonsignifcant difference in the *β*‐coefficient of MAD only.

## 4 DISCUSSION

In parallel with unprecedented political commitment to improve breastfeeding and complementary feeding practices and reduce chronic child undernutrition, Nepal made considerable progress on IYCF and child linear growth faltering over the 1996–2016 period. Of the eight WHO core IYCF indicators, EIBF, INTRO, MAD, and IRON improved, whereas EBF and continued breastfeeding at 1 year experienced minor declines. Although bottle‐feeding increased substantially, the majority of the seven WHO optional IYCF indicators and the three additional IYCF indicators increased gradually over the past two decades. Furthermore, our findings indicate rapid and sustained improvements in HAZ and a reduction in the prevalence of stunting from 1996–2016. Nevertheless, there remains a scope to further improve age‐appropriate IYCF practices in Nepal (Benedict et al., 2018; Na et al., 2018). To illustrate, for Nepal to attain a *very good* rating for all five required IYCF indicators in the WHO IYCF assessment tool, the prevalence of EIBF, EBF, and INTRO need to increase by 34, 24, and 18 p.p., respectively, and the prevalence of bottle‐feeding must drop by 9 p.p. to a rate commensurate to that in the mid‐1990s (WHO, 2003). Our findings are in line with Nepal's Global Breastfeeding Scorecard, which stated that the country was ranked only fourth among the eight South Asian countries (Afghanistan, Bangladesh, Bhutan, India, Maldives, Nepal, Pakistan, and Sri Lanka) for EIBF, second for EBF, but first for both continued breastfeeding at 1 and 2 years in 2016 (WHO & UNICEF, 2017). EIBF is reported to improve breastfeeding success, health and survival of neonates, and the emotional attachment of the child to their mother (Debes, Kohli, Walker, Edmond, & Mullany, 2013). However, prelacteal feeding is known to disrupt EIBF and is a major barrier to EBF by delaying the onset of breastfeeding and milk arrival (Benedict et al., 2018; Bhandari et al., 2019). In Nepal, there is a preference for prelacteal feeding, which includes honey, ghee (refined butter), sugar water, and animal milk (Khanal, Adhikari, Sauer, & Zhao, 2013; Khanal, Scott, Lee, Karkee, & Binns, 2015). Furthermore, maternal perception of insufficient breastmilk supply and sociocultural norms (Gatti, 2008; Pries et al., 2016) are recognised as pervasive barriers to EBF (Patel et al., 2015; Sundaram et al., 2013).

IYCF has been increasingly recognised as a critical driver of improved child nutrition (Black et al., 2008), and recent evidence has shown associations between age‐appropriate IYCF practices and child linear growth outcomes (Aguayo, Nair, Badgaiyan, & Krishna, 2016; Jones et al., 2014; Krasevec, An, Kumapley, Bégin, & Frongillo, 2017; Lamichhane et al., 2016; Menon, Bamezai, Subandoro, Ayoya, & Aguayo, 2015). However, results are highly varied and more country‐specific research is needed. Our regression–decomposition analyses first indicated that a paucity of IYCF indicators—including INTRO, MDD, minimum meal frequency, MAD, predominant breastfeeding, and consumption of ASF—are associated with improved child linear growth outcomes, and second, the statistical contributions of age‐appropriate IYCF practices on long‐term age‐specific increases in HAZ and reductions in stunting are modest. Hence, our findings support the notion that the aetiology of child linear growth faltering is complex and multifactorial (Headey et al., 2017; Menon et al., 2018), and improving IYCF in isolation is insufficient to ensure further progress against chronic child undernutrition. Our findings also mirror previous studies that have indicated the critical role of MDD—reflecting micronutrient density adequacy of complementary foods—in relation to child linear growth outcomes (Arimond & Ruel, 2004; Jones et al., 2014; Menon et al., 2015). Nevertheless, child dietary diversity and diet quality remains a persistent concern in Nepal. Cunningham, Headey, et al. (2017) reported that solid or semi‐solid complementary foods in Nepal consisted mainly of nutrient‐poor rice, jaulo (rice and lentil porridge), soup, lito (roasted cereal and lentil porridge), Cerelac, and fruit juices, although ASF tended to be introduced only after 12 months of age (Cunningham, Headey, et al., 2017). Furthermore, there are widespread cultural beliefs that cereals are sufficient for child nutrition, with little emphasis on the importance of MDD or IRON for child linear growth and development (Gautam, Adhikari, Khatri, & Devkota, 2016). Moreover, ASF, green leafy vegetables, fruits, and yoghurt are generally not given to young children in Nepal due to cultural taboos and the common perception that these foods are difficult to digest (Locks et al., 2015; Siegel et al., 2006). Similar to recent studies, our findings indicate that consumption of ASF is associated with improved child linear growth outcomes (Headey, Hirvonen, & Hoddinott, 2018; Krasevec et al., 2017; Shapiro et al., 2019). However, the lack of sensitivity and specificity of many IYCF indicators might contribute to the relationships observed in our analyses. Therefore, stronger proxies of diet quality and quantity are warranted to elucidate how age‐appropriate IYCF practices relate to child linear growth faltering over time (Jones et al., 2014).

Our findings on the lack of association between breastfeeding indicators and HAZ or stunting—and subsequent limited statistical contribution to the progress over time as compared with complementary feeding—should not be interpreted as breastfeeding being unimportant for child survival, growth, and development (Darmstadt et al., 2005; Jones, Steketee, Black, Bhutta, & Morris, 2003; Kramer & Kakuma, 2012). To illustrate, associations between EBF and child linear growth outcomes are often only apparent in later childhood because nutrition outcomes such as stunting are cumulative by nature and do not fully set in until the second year of life (Victora et al., 2010). Nevertheless, a review by Bhutta et al. (2008) showed that strategies for breastfeeding promotion have only small effects on child stunting.

The significant associations between various IYCF indicators and child linear growth outcomes reiterate the public health importance of age‐appropriate IYCF practices and advocate for policies, programmes, healthcare systems, workplaces, and communities that protect, promote, and support IYCF. Government leadership and investment, alongside contributions at all levels of society, are critical to drive future IYCF and child linear growth improvements (Rollins et al., 2016). At present, action to stimulate investment in IYCF is gaining momentum under the Global Breastfeeding Advocacy Initiative, under leadership from UNICEF and WHO, and the “First Foods” global meeting (UNICEF, 2016). Moreover, multiple long‐term national actions championing optimal IYCF exist in South Asia (Thow et al., 2017). To illustrate, the Government of Nepal passed the Mother's Milk Substitutes Act in 1992 (Ministry of Health and Population Nepal, 1992), and Nepal's Ministry of Health and Population trained over 80 breast milk substitute monitors to control adherence to the breast milk substitute marketing code in 2018. Furthermore, Nepal's National Nutrition Policy and Strategy 2004, its amendment in 2008, and the Multi‐Sectoral Nutrition Plan I and II (2018–2022) have given special emphasis on optimal IYCF for improved child nutrition, including strengthened monitoring and accountability within a lifecycle approach starting with the first 1000‐day period (Ministry of Health and Population Nepal, 2008, 2017). In addition, the national Child Grant, Ten Steps of the Baby‐Friendly Hospital Initiative, and “Golden 1000 Days” public awareness campaigns via mass media might also have impacted the observed progress on IYCF in Nepal (Karn, Devkota, Uddin, & Thow, 2017; WHO & UNICEF, 2009).

The progress on age‐appropriate IYCF practices over the past two decades might also signify the scale‐up and adequate coverage of nutrition/IYCF programmes during Multi‐Sectoral Nutrition Plan I and II. To illustrate, the community‐based Suaahara project and integrated Micronutrient Powder program directly advocated and improved IYCF practices (Cunningham, Singh, et al., 2017; Locks et al., 2018). Furthermore, childhood and neonate illness management programs, such as the Community Based Integrated Management of Childhood Illness and Community Based Newborn Care Program, also focused on improving IYCF in Nepal (Dawson et al., 2008; Paudel, Shrestha, Siebeck, & Rehfuess, 2017). In addition, prenatal IYCF education and counselling and frontline health professionals have played imperative roles to encourage mothers to adopt age‐appropriate IYCF practices (Rosen, Krueger, Carney, & Graham, 2008; Swanson & Power, 2005). Nevertheless, Paudel, Basaula, and Tiwari (2018) and Cunningham, Headey, et al. (2017) report considerable maternal knowledge gaps with regard to age‐appropriate IYCF practices. Lastly, the Reproductive Health Bill in 2018 and policy support for workplace crèches, breastfeeding breaks in line with ILO Convention 183, and breastfeeding rooms might ensure than IYCF and work are not mutually exclusive in Nepal (Thow et al., 2017). Positive national‐level changes in IYCF practices and child linear growth outcomes might also reflect improved socioeconomic status, parental education, and/or sanitation, as well as exposure to and utilisation of healthcare‐related services (Cunningham, Headey, et al., 2017; Headey & Hoddinott, 2015).

Interpretations of causality in our research are limited by the use of cross‐sectional data, potentially confounded by omitted variables. Furthermore—although infant and young children's diets are often monotonous in low‐ and middle‐income countries (Ruel, 2003)—age‐appropriate complementary feeding practices were based on a single 24‐hr recall per child—introducing random intraperson error—which might not be representative of “usual” dietary intake and thus attenuate our *β*‐coefficients (Thorne‐Lyman, Spiegelman, & Fawzi, 2014). Hence, the directions of the relationships observed are only hypothesised, that is, age‐appropriate IYCF practices are positively associated with child linear growth outcomes. The causal sequence of these relationships cannot be determined for the available data. However, the plausibility of our findings are strengthened by the adjustment for several potential nutrition‐sensitive and nutrition‐specific confounders—hypothesised to be associated with child linear growth—and time‐invariant controls. Furthermore, our analyses used the most comprehensive and highest quality national survey data sets for five time points between 1996 and 2016 in Nepal. The findings are also largely consistent with theoretical expectations and previous studies on the topic. Examining the associations between our model time‐variant covariates and child linear growth outcomes was beyond the scope of this study.

## 5 CONCLUSIONS

This is the first statistical decomposition to comprehensively investigate the contributions of the spectrum of IYCF indicators on age‐specific HAZ and stunting prevalence over time. Long‐term trends suggest substantial improvements in IYCF practices and child linear growth over the past two decades in Nepal. Nevertheless, our findings also indicate that only a few IYCF indicators—in particular complementary feeding—are significantly associated with improved child linear growth outcomes. Moreover, age‐appropriate IYCF practices, in isolation, have modest contributions to the rapid and sustained reduction in age‐specific child linear growth faltering from 1996–2016 in Nepal. However, given the inextricable link between successful breastfeeding and complementary feeding, it important to aim to improve the entire continuum of IYCF practices in the first 2 years of life. Our findings indicate that multisectoral nutrition strategies that include approaches to protect, promote, and support IYCF are critical to further accelerate the progress against chronic child undernutrition in Nepal. Furthermore, specific focus is needed to improve age‐appropriate IYCF practices that have shown no significant development over the past two decades, including EBF, MAD, and minimal bottle‐feeding.

## CONFLICTS OF INTEREST

P.D. and S.C. are UNICEF staff members. The opinions and statements in this article are those of the authors and may not reflect official UNICEF policies. The authors declare that they have no conflicts of interest.

## CONTRIBUTIONS

G.H‐C., A.A., and P.K. performed the research. G.H‐C., A.A., P.D., and P.K. designed the research study. G.H‐C. and A.A. analysed the data. G.H‐C., A.A., P.D., and S.C. wrote the paper.

## Supporting information


**Figure S1.** **Shifts in distribution of height‐for‐age z‐score (HAZ), 1996 to 2016.** Source: Kernel density estimates from Nepal's Family Health Survey 1996 and Nepal demographic and Health Survey 2016

Table S1. Independent variable definitions.

## Data Availability

All our data are based on Demographic and Health Surveys, which are available at the Measure DHS website after appropriate registration: http://dhsprogram.com/data/available‐datasets.cfm.
