# Supplementary material for: Infant and young child feeding practices and child linear growth in Nepal: Regression–decomposition analysis of national survey data, 1996–2016
Source: Matern Child Nutr. 2020 Jan 10;18(Suppl 1):e12911. doi: 10.1111/mcn.12911 (PMC8770650; doi:10.1111/mcn.12911)
Supplement: Supplementary file 2 — Table S1. Independent variable definitions. [file MCN-18-e12911-s002.docx]

**Table S1. Independent variable definitions.**

| **Short name** | **Definition** |
| --- | --- |
| Asset index (1-10) | 5-component index^†^ |
| Maternal education (years) | Mother’s years of education |
| Paternal education (years) | Father’s years of education |
| Prenatal doctor visit (0/1) | Dummy = 1 if mother received a prenatal visit from a doctor |
| 4^+^ ANC visits (0/1) | Dummy = 1 if mother received 4 or more antenatal care (ANC) visits |
| Iron during pregnancy (0/1) | Dummy = 1 if mother received iron supplements during pregnancy |
| Born in medical facility (0/1) | Dummy = 1 if child was born in hospital or other medical facility |
| Maternal BMI (kg/m²) | Mother’s body mass index |
| Maternal height (0/1) | Dummy = 1 if mother’s height ≥ 145cm |
| All vaccinations (0/1) | Dummy = 1 if child received BCG; polio (2 shots); diphtheria; pertussis and tetanus (3 shots); and measles vaccines between 1996-2011 and/or pentavalent vaccination (3 shots) in 2016 |
| Birth order (rank) | Order a child is born in the family |
| Preceding birth interval (months) | Interval between birth of present child and any previous child |
| Open defecation (0/1) | Dummy = 1 if household does not have access to a latrine or toilet |
| Water-tube well (0/1) | Dummy = 1 if household drinking water was sourced from tube well |
| Water source-piped (0/1) | Dummy = 1 if household drinking water was sourced from pipes |

Source: Authors’ construction. ^†^Pooled principal components analysis (PCA): bicycle ownership, television ownership, radio ownership, non-natural flooring, and household access to electricity.

**
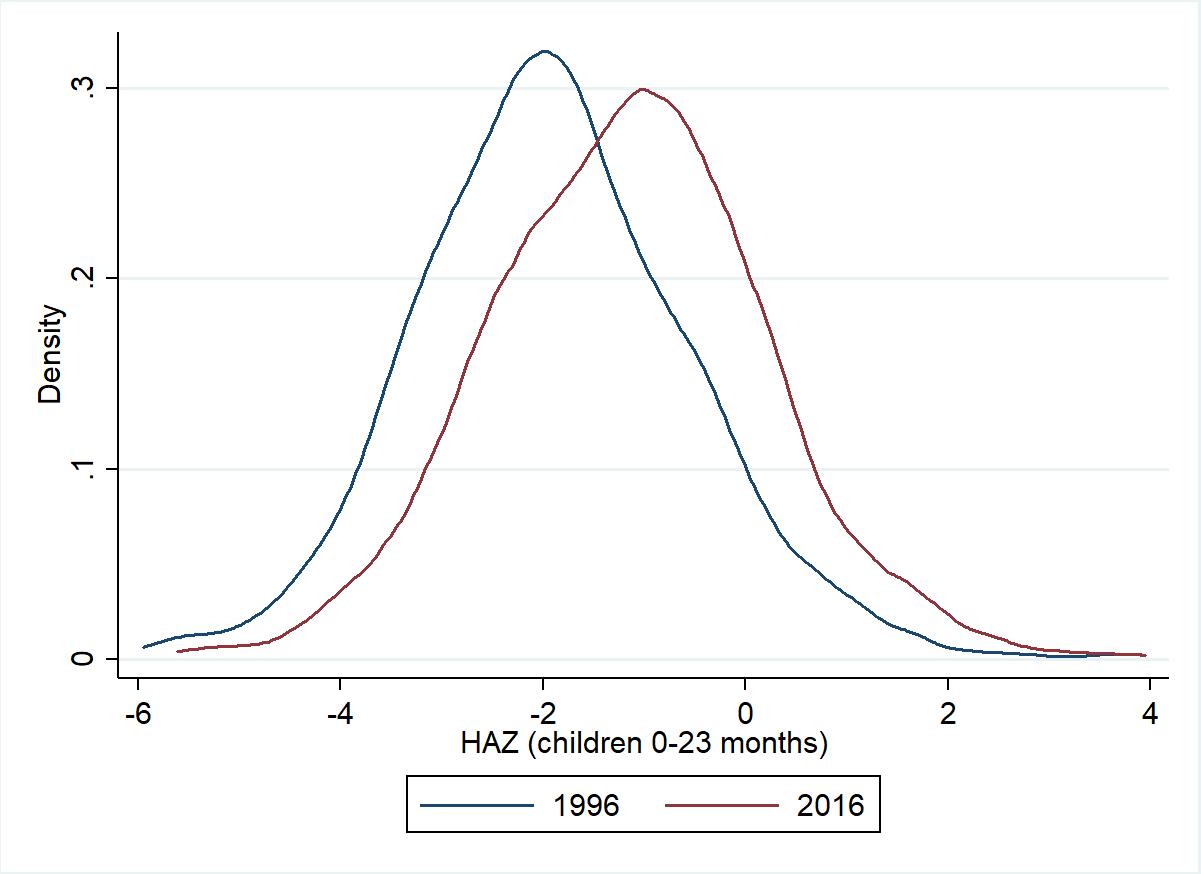
**

**Figure S1. Shifts in distribution of height-for-age z-score (HAZ), 1996 to 2016.** Source: Kernel density estimates from Nepal’s Family Health Survey 1996 and Nepal demographic and Health Survey 2016
